# 

**Published:** 1887-11

**Authors:** 


### CONTENTS.

### COMMUNICATIONS.

Woman in Dentistry........ 525
Monograph of the First Permanent Molar........ 529

### PROCEEDINGS.

Ninth International Dental Congress, Washington, D. C., Sept., 1887.. 535

### SELECTIONS.

Removal of Right Half of Lower Jaw by Slow Enucleation........ 564
A Move in the Right Direction........ 565
Warm Ether as an Anæsthetic........ 567
A Case of Tetanus Cured by Hypodermic Injections of Cocaine...... 567
Prominence of the Metric System........ 568
Read Medical Journals........ 568
The Human Nose is a Wonderful Instrument........ 569
Increase of Blindness in the United States........ 569
Iron Lemonade........ 570
Female Medical Students........ 570
SALMAGUNDI........ 571
Treatment of Stomatitis........ 573

### EDITORIAL.

The Ohio State Dental Society........ 574
The Congress........ 574
An Unusual Opportunity........ 575
In Memoriam........ 575

